# Antioxidant capacity sources of soils under different land uses

**DOI:** 10.1038/s41598-024-58994-9

**Published:** 2024-04-10

**Authors:** Irmina Ćwieląg-Piasecka, Jacek Łyczko, Elżbieta Jamroz, Andrzej Kocowicz, Dorota Kawałko

**Affiliations:** 1 Institute of Soil Science, Plant Nutrition and Environmental Protection, Wroclaw University of Environmental and Life Sciences, Grunwaldzka 53 St., 50-357 Wroclaw, Poland; 2 Department of Food Chemistry and Biocatalysis, Wrocław University of Environmental and Life Sciences, Norwida 25, 53-375 Wrocław, Poland

**Keywords:** Environmental chemistry, Environmental impact, Environmental sciences

## Abstract

Antioxidants (AOX) in soils originate mainly from secondary plant metabolites and are pivotal in many redox processes in environment, maintaining soil quality. Still, little is known about the influence of land uses on their accumulation in soil. The aim of the paper was to determine the content of these redox-active compounds in the extracts of A horizons of abandoned fallows, arable and woodland soils. Total antioxidant capacity (TAC) of soils under various uses and vegetation was evaluated in different soil extracts using Folin-Ciocâlteu method. The contribution of humic acids to TAC was determined and antioxidant profiles estimated using the chromatographic GC–MS method. Forest soils exhibited the highest TAC (15.5 mg g^−1^) and AOX contents (4.34 mg g^−1^), which were positively correlated with soil organic carbon content. It was estimated that humic acids contribute to over 50% of TAC in soils. The main phenolics in woodland A horizons were isovanillic and p-hydroxybenzoic acid (p-HA), while esculetin and p-HA predominated in the abandoned fallows due to the prevalence of herbaceous vegetation. Cultivated soils were the most abundant in p-HA (56.42%). In the studied topsoils, there were considerable amounts of aliphatic organic matter, which role in redox processes should be further evaluated.

## Introduction

Antioxidants (AOX) are defined as substances present most often in low concentrations when compared to easily oxidizable substrates that delay or inhibit their oxidation^1^. They occur naturally in soils as products of plant metabolism, including enzymes, hormones, amino acids, vitamins or polyphenols, and are specific to the land vegetation profile^2–4^. AOX may also originate from industrial products or wastes, which enter the soil either as leachates or as particulate matter^5,6^. Some of the low-molecular-weight antioxidants in soil, namely phenolic compounds (PC, phenolics), are the most abundant group of secondary plant metabolites exuded from the roots or grains^7,8^. The PC family covers a broad spectrum of chemical compounds, such as phenolic acids (derivatives of benzoic and cinnamic acids), flavonoids (flavones, flavanols, flavonols, isoflavones and anthocyanins), tannins, coumarins, lignans, quinones, stilbenes, curcuminoids etc^9^. They contribute to the adjustment of the plant microbiota to soil conditions, modulate its biodiversity and participate in many redox reactions, including inhibition of free radicals as well as control of plant residue biodegradation and nutrient cycling^10,11^. The PC content of soil, together with its antioxidant capacity, is considered an “antioxidant system”, which was proposed as an indicator of its health and quality^11^.

In the soil environment, phenolic compounds are commonly generated during the decomposition of soil organic matter (SOM), based on the physical breakdown and biochemical transformation of complex organic molecules into simpler organic and inorganic moieties^12^. In parallel, these processes lead to the formation of humic substances (HS) in soil, containing stable semiquinone radicals^13^ in their structure, most likely to be derived from the reaction of phenolic compounds with reactive radicals^14^. Several studies imply that most of the antioxidants in soil can be found among the humic substances^15–18^. This was evaluated by Ziółkowska et al^19^., who determined the important role of phenolic compounds in the processes of organic matter transformations in meadow soils, leading to the formation of humic substances. What is more, phenolics are considered more resistant to decomposition in soils compared to other organic matter sources, and thus have often been regarded a slow carbon pool in soil dynamics models^6^. Therefore, the soil's antioxidant capacity may control the rate of breakdown of more labile organic matter pools and the recalcitrance of SOM^15^.

Despite the important role of phenolic compounds in many processes in soils, the literature on the antioxidant capacity of soils is still fragmentary and relates mostly to lands under agricultural use. The studies by Rimmer^20^ demonstrated that antioxidants can be extracted from soils and their quantity varies from soil to soil, decreasing with the depth of the soil profile^14,15^. This effect was correlated with the diversity of plant residues and their phenolic compositions that were incorporated into the soil material. Huge differences were also found between the amounts of individual phenolic compounds, depending on the land use^20^, although majority of studies concerning AOX were performed on cultivated soils^21^. Cardelli et al^11^. compared the antioxidant capacity of agricultural, naturally grazed, and forest soils in the Mediterranean zone and found that it was the highest in grasslands as related to the amount of alkali-soluble phenols^3^. Antioxidant properties of soils were also investigated in the polar region by Shamrikova et al^22^. They assigned the low AOX content in cryogenic soils to the low productivity of tundra plant communities, and correlated with the enhanced sensitivity of polar ecosystems to climate change. This was also highlighted in the studies of Min et al^6^., who proposed to use phenolics as a tool to control rates of SOM decomposition to further stabilize organic carbon in the environment and increase soil’s quality. In turn, soil typology as well as organic carbon and nitrogen contents, were identified as important factors determining antioxidants concentration in many crops. Oney-Montalvo et al^23^. found that the chemical composition of black soils enhanced its enzymatic activity and stimulated the biosynthesis of polyphenols in the habanero peppers, resulting in their increased total polyphenols content and antioxidant activity measured with the DPPH assay.

Several methods of extraction and quantification of phenolic compounds in soils have been evaluated to date^6^. Currently, all of them require the preparation of soil extract covering all potentially available antioxidants, not only the pool of free phenolics that are easily soluble in water. Insoluble AOX in soil may be present bound to recalcitrant macromolecules of SOM; therefore, alkaline extraction is commonly used^3,11,20^. Recently, Ziółkowska et al^24^. proposed an isolation method for phenolic compounds that comprises two stages: acid hydrolysis followed by alkaline re-hydrolysis. It was found to be an efficient extraction method for insoluble-bound forms of polyphenols from plant and soil material, although up to date only meadow soils have been tested. Meanwhile, in the literature several methods have been suggested to determine the phenolics content or antioxidant capacity of various extracts, based on either reduction of metal ions using a tested antioxidant or scavenging of stable radicals^17,18,20^. Among them, the Folin–Ciocâlteu (FC) colorimetric assay has been commonly proposed to determine the total phenolics mainly in various plant-derived materials^6,18,25^. The FC reaction is based on single electron transfer, and measures the reductive capacity of a mixture containing redox-active phenolic compounds^26^. However, what should be kept in mind is that different matrices may impair the assay’s accuracy, as the FC reagent can also be reduced by amino acids and proteins, although the share of the process is postulated to be less significant compared to the reduction by phenolics^27^. Therefore, the FC method determines the total antioxidant capacity (TAC) of the sample rather than solely the total phenolics (TPs) content. In addition, the assay is relatively simple and less expensive compared to the other techniques, including chromatographic methods^28^, although they further allow the qualitative analysis of the tested mixtures (phenolic profiles). Thus, it may serve as a universal test for the determination of the AOX potential of soils or for monitoring purposes in the evaluation studies of soil quality.

The current literature on phenolics content is devoted mostly to plants or plant-derived products and, up to date, a limited number of papers concerning AOX content in soils have been published^14,15,20–22,24^. To the best of our knowledge, no such studies have been performed on abandoned fallow lands, where, due to the undisturbed succession, various organic matter transformations may lead to the accumulation of organic carbon in soil influencing its antioxidant system and soil’s quality. Therefore, the specific objective of the study was to compare the total antioxidant capacity (TAC) of soils under various land uses, namely abandoned fallows, arable and forest soils and identify their phenolics profiles. In addition, the study aimed to estimate the relative share of humic acids in antioxidant potential of investigated soils, and evaluate the efficiency of various AOX extraction methods proposed in literature.

## Materials and methods

### Chemicals used

Sodium hydroxide (NaOH), hydrochloric acid (HCl), ascorbic acid, sodium carbonate (Na_2_CO_3_) and ethylenediaminetetraacetic acid (EDTA) were of analytical grade, while ethyl acetate was of HPLC grade; all purchased from Avantor Performance Materials (previously POCH, Poland). Folin-Ciocâlteu reagent, pyridine, N,O-bis(trimethylsilyl)trifluoroacetamide (BSTFA), analytical standards of phenolic compounds: gallic and caffeic acids, as well as fine granular quartz from Sigma-Aldrich (Steinheim, Germany). The water was purified using the Millipore Milli-Q system.

### Soil sampling and analysis

The research was carried out in three rural regions of SW Poland, located near Radomierz, Lubin, and Wrocław, indicated as R1, R2, and R3, respectively. Soil samples for the studies were collected in triplicate from abandoned fallows (O), arable lands (P) or woodlands (L, Table 1). In the group of soils taken out of cultivation one of the plots (O1) was abandoned for agricultural production in the 1970s due to the difficulty of access, while the other plots (O2-O4), which had been used to grow mainly rye and potatoes, were abandoned for agricultural production in the 1990s because of low productivity.

**Table 1:** Soil location, land use and classification.

Region	Profile number	Land use	Habitat type	Vegetation	Soil type (WRB 2014)
R1	O1	Abandoned fallow	Natural meadow	*Dactylis glomerata, Stellaria media, Vicia, Aegopodium podagraria*	Gleyic Cambisol
	P1	Arable land	Rape field	*Brassica napus L. var. napus*	Dystric Cambisol
	L1	Woodland	Mixed forest	*Picea A. Dietr., Quercus L., Acer L., Alnus glutinosa L. Gaertn., Urtica dioica*	Dystric Cambisol
R2	O2	Abandoned fallow	Natural meadow	*Solidago virgaurea, Sonchus arvensis, Trifolium pratense, Centaurea cyanus L.,*	Gleyic Fluvisol
	P2	Arable land	Corn field	*Zea mays*	Gleyic Cambisol
	L2	Woodland	Mixed forest	*Pinus nigra, Robinia pseudoacacia L., Quercus L., Acer L., Betula pendula*	Brunic Arenosol
R3	O3	Abandoned fallow	Natural meadow	*Artemisia vulgaris, Achillea millefolium L., Sonchus arvensis, Calamagrostis epigejos (L.) Roth*	Gleyic Fluvisol
	P3	Arable land	Rye field	*Secale L*	Gleyic Fluvisol
	L3	Woodland	Mixed forest	*Pinus nigra, Betula pendula, Fagus sylvatica, Acer L*	Haplic Podzol
	O4	Abandoned fallow	Natural meadow	*Calamagrostis epigejos (L.) Roth, Centaurea cyanus L., Taraxacum officinale, Artemisia vulgaris*	Gleyic Fluvisol
	P4	Arable land	Rye field	*Secale L*	Gleyic Fluvisol
	L4	Woodland	Mixed forest	*Quercus L., Acer L., Betula pendula, Pinus nigra, Fagus sylvatica*	Gleyic Fluvisol

Soil materials from four abandoned fallow soils (O1–O4), four cultivated soils (P1–P4), and four forest soils (L1–L4) were analyzed in the study in triplicate, giving a total of 36 samples. For antioxidant studies, three subsamples of each plot, taken from a dozen points, were collected from A horizons, analyzed separately, and the results expressed as their mean. Additionally, soil stripping was carried out for the studied soils and materials for further laboratory analyses were collected from all morphologically distinguished horizons and subhorizons to describe soils according to FAO-WRB classification^29^.

The soil samples were dried at room temperature, ground, sieved (2 mm), and subjected to the following analyses:

- Total organic carbon (TOC) and total nitrogen (TN) were measured using a Vario Macro Cube CN analyser (Elementar Analyser System GmbH, Germany).
- Soil pH was measured potentiometrically in a distilled water and 1 M KCl suspensions at the soil:water ratio of 1:5 (v/v) (Mettler Toledo, Columbus, OH, USA).
- Calcium carbonate content was assessed volumetrically using Scheibler apparatus^30^;
- Particle size distribution was conducted by sieve and sedimentation method and estimated texture classes were assigned according to the USDA standard applied by the WRB classification.

The texture and the chemical composition of topsoil samples investigated in this study were given in Table 2. None of the investigated soils contained a measurable calcium carbonate level. Soil materials differed in organic carbon content, texture, and pH.

**Table 2:** Selected physicochemical properties of topsoils (A horizons) under study. Results are expressed as the mean values ± standard deviation (*n* = 3).

Sample	TOC (%) ± SD	TN (%) ± SD	C/N	pH _H2O_ ± SD	pH _KCl_ ± SD	Texture*
O1A	2.52 ± 0.38	0.22 ± 0.09	11.45	5.02 ± 0.25	3.49 ± 0.21	SiL
O2A	1.61 ± 0.25	0.05 ± 0.01	32.20	4.68 ± 0.22	3.68 ± 0.19	S
O3A	0.88 ± 0.07	0.03 ± 0.01	29.33	6.09 ± 0.23	5.38 ± 0.30	S
O4A	0.84 ± 0.12	0.04 ± 0.01	21.00	4.13 ± 0.42	3.60 ± 0.36	S
P1A	1.88 ± 0.23	0.14 ± 0.03	13.43	5.62 ± 0.02	4.42 ± 0.06	SiL
P2A	1.01 ± 0.11	0.08 ± 0.01	12.63	5.79 ± 0.23	4.94 ± 0.22	SL
P3A	0.73 ± 0.17	0.05 ± 0.02	14.60	4.28 ± 0.16	3.53 ± 0.19	S
P4A	0.70 ± 0.13	0.04 ± 0.02	17.50	5.35 ± 0.21	4.89 ± 0.27	S
L1A	2.27 ± 0.41	0.16 ± 0.05	14.18	4.56 ± 0.37	3.39 ± 0.19	SiL
L2A	1.30 ± 0.51	0.04 ± 0.03	32.50	3.74 ± 0.20	3.39 ± 0.29	LS
L3A	1.16 ± 0.09	0.04 ± 0.02	29.00	3.72 ± 0.25	3.35 ± 0.32	LS
L4A	2.14 ± 0.25	0.12 ± 0.01	17.83	5.12 ± 0.37	4.62 ± 0.24	LS

* SiL—silt loam, S—sand, SL—sandy loam, LS—loamy sand.

## Extraction of phenolic compounds

### Water and alkaline extraction

Water and alkaline extracts were prepared by mixing 5 g of each soil sample with 50 mL of water or 0.1 M NaOH, respectively, and agitating on a rotary shaker for 4 h (Biosan, Multi RS-60). Subsequently, the mixtures were left overnight and centrifuged at 4500 rpm for 5 min. Additionally, to assess the share of humic acids (HA) in the antioxidant potential of the extract, the alkaline extracts were acidified to pH 1 with 6 M HCl, left overnight, and the precipitated humic acids centrifuged and discarded (4500 rpm, 20 min). Therefore, three types of supernatants were obtained: water (WE), alkaline (AlE) and acidified (AcE, with HA removed). All of them were subjected to a total antioxidant capacity (TAC) assessment with the use of UV–Vis spectroscopy.

## Acid hydrolysis and alkaline re-hydrolysis of soil samples (AAH extracts)

In addition, the extraction of phenolic compounds from soil samples described in Ziółkowska et al^24^. with some modifications, was also tested. Briefly, to 1 g of soil sample, 2 mL of ascorbic acid (1%), an aliquot of 0.0125 mM EDTA and 10 mL of 6 M HCl were added (acid hydrolysis, step I). Ascorbic acid and EDTA were utilized to protect the phenolics from degradation. It was then followed by the sample microwave extraction at 120 °C for 2 h (650 W, microwave system StartD, Milestone, Sorisole, Italy). A similar procedure was proposed by Martens^31^ during the hydrolysis of ether-linked phenolic acids from soil, but the temperature was higher–160 °C. After cooling, the supernatants were transferred to the amber glass vials and kept in the fridge. The soil remnants were neutralized with ultrapure water, and 2 mL of ascorbic acid (1%), 2 mL of 0.0125 mM EDTA and 10 mL of 10 M NaOH were added, followed by 24 h of agitation (alkaline re-hydrolysis, step II). After centrifugation at 10 000 rpm for 5 min the supernatants were acidified to pH 1, using 6 M HCl. The mixtures were left overnight, and precipitated humic acids were centrifuged (4500 rpm, 20 min) and discarded. As a result, hydrolysates soluble in acid remained. The supernatants obtained in the alkaline re-hydrolysis step were combined with the solutions from acid hydrolysis and labelled as AAH. They were further analyzed for the total antioxidant capacity (TAC) on UV–Vis and the phenolic profile estimation using GC–MS. In addition, pure quartz was subjected to an identical procedure to exclude the influence of ascorbic acid on the results of the FC assay.

### Estimation of the Total Antioxidant capacity (TAC) using Folin- Ciocâlteu method.

The Folin-Ciocâlteu (FC) reaction is an antioxidant assay based on electron transfer, which measures the reductive capacity of the sample^32,33^. The reagent utilized in the method is a mixture of sodium tungstate, sodium molybdate, lithium sulfate, bromine water, and concentrated hydrochloric and phosphoric acids. The resulting product is a blue colored complex, and the absorbance of the sample is measured within the wavelength range of 750–784 nm^34^. The method has been widely applied to determine the total phenol/polyphenol content of plant-derived food and biological samples^26^, in which the product of phenolics oxidation by the FC reagent is measured at 765 nm. In the presented paper, the assay was adopted to test the antioxidant potential of various extracts from the studied soil samples.

The total antioxidant capacity of water extracts (containing readily available phenolics) was determined according to the following procedure: 5 mL of extract was mixed with 0.5 mL of FC reagent and, after 3 min, 1.5 mL of 20% Na_2_CO_3_ was added^35^. The mixtures were then allowed to stand for 30 min in the dark, and in the next step, the absorbance of the solutions was measured at 765 nm using a Cary 60 UV–Vis spectrophotometer (Agilent, Santa Clara, CA, USA). The calibration curve used in the quantification was drawn up for gallic acid solutions in the concentration range 1–10 mg L^−1^. For the alkaline (0.1 M NaOH before and after acidification) and AAH types of extracts 0.4 mL of the sample was taken up, 7.8 mL of water and 0.5 mL of FC reagent were added, followed by the addition of 1.5 mL of 20% Na_2_CO_3_. The rest of the procedure was analogous to the one described for water extracts. A calibration curve in the range of 0–500 mg L^−1^ of gallic acid equivalents was constructed (Figure S1). Bastola et al^28^. proved that gallic acid as a standard is the most appropriate in the FC assay of total phenolic content quantification compared to other conventionally used phenolic acids.

### Phenolics profiles in AAH extracts

The qualitative and quantitative analysis of antioxidants present in the studied soils was conducted on the AAH extracts, as their TAC assessed by the FC method was the highest. The method described by Pachura et al^36^. was used to determine the AOX profiles. Briefly, 1 mL of soil extract was taken and 3.5 µg of caffeic acid was added as an internal standard. Subsequently, the sample was extracted three times with 1 mL aliquots of ethyl acetate. The resulting extracts were combined and subjected to solvent evaporation using a vacuum evaporator. The dry extract was dissolved in 250 µL of pyridine, 250 µL of BSTFA was added and silylation was carried out for 45 min at 60 °C. The sample was then transferred to a 1.5 mL chromatographic vial and subjected to GC–MS analysis.

GC–MS analysis was carried out using the Shimadzu GCMS QP 2020 instrument (Shimadzu, Kyoto, Japan) with a Zebron ZB-5 MSi column (30 m × 0.25 mm × 0.25 μm; Phenomenex, Torrance, CA, USA). Injection volume of 1 µL was performed at 280 °C, split 10, carrier gas flow (helium) 0.97 mL min^−1^. The separation of analytes was performed using the following temperature program: 150 °C for 1 min, raised to 300 °C at a rate of 10 °C min^−1^ and maintained at 300 °C for 5 min. Mass analysis was performed with the following parameters: ion source temperature of 270 °C, an interface temperature of 270 °C and a scan mode of 40–800 m/z. Identification of the analytes was performed based on the NIST 20 database (National Institute of Standards and Technology). Quantification was carried out by peak area normalization to an internal standard^37,38^.

### Statistical analysis

The normal distribution of the data was checked using the Shapiro–Wilk test. Basic statistical parameters such as the mean, standard deviation (SD), and standard error (SE) were calculated based on the triplicate result. The values of total antioxidant capacities (TAC) obtained for soils under different land uses, as well as the results of phenolics profiles were statistically compared using the Student’s t-test (p < 0.05). Pearson’s correlation was used to assess the strength of the dependence of estimated TAC values for various extract types on the TOC of the investigated horizons and the sum of individual antioxidants (SUM) identified with GC–MS in the studied soils.

All the data were processed using the software package Statistica 13.3 TIBCO Software Inc.

## Results and discussion

### Total antioxidant capacity (TAC) of the investigated soils.

Results of the Folin-Ciocâlteu assay of all the tested extract types (in µg mL^−1^), expressed as the total antioxidant capacity of the investigated soils (in µg g^−1^), were collected in Table 3. The readily available, dissolved forms of phenolic compounds (aqueous extracts, WE) made up only a small part of the potential pool of antioxidants in the topsoil horizons of the studied soils. However, their role should not be marginalized, as these compounds, present by definition in low concentrations in soil solution, participate in many redox processes, including the formation of humic substances^19^. TAC in WE ranged from 5 to slightly over 21 µg g^−1^ for abandoned soils and farmlands to about 114 µg g^−1^ for forest soils. Meanwhile, alkaline extraction of soil material (AlE) resulted in 30 to even 56 times larger TAC (on average) compared to WE. It can be attributed to the high efficiency of NaOH as a solvent, which releases bound and polymerized forms of phenolics from soil^6^. The maximum TAC estimated for AlE was obtained for forest soils, achieving up to 2228.67 µg of gallic acid equivalents per g of soil, whereas the lowest value was found for cultivated soils (135 µg g^−1^). Mean TAC values, based on the AlE, point to the following decreasing order of antioxidant potential: forest soils > abandoned fallows = arable soils. Thus, the A horizon of forest soils is characterized by a higher antioxidants content than that of abandoned or cultivated soils, which is the consequence of an enhanced accumulation of fresh organic matter in the forest floor^39^. The obtained results are in contradiction to the studies of Cardelli et al^3^*.,* who found the higher antioxidant capacity in Mediterranean soils of grasslands rather than forests. However, the discrepancy in trends obtained between the results of Cardelli and those presented herein might result from the various climatic zones and thus, the specificity of vegetation and soil dynamics under which the AOX were accumulated. When comparing the mean TAC values for soils taken out of cultivation and farmlands, it can be elucidated that their antioxidant potential is of similar magnitude. Nevertheless, a great deal of variability within the soils of each land use and their TAC should be noted (Table 3). This can be most likely attributed to differences in the density and diversity of plant cover that is prone to mineralization and humification on various land types^40^.

**Table 3:** Summary of the total antioxidant capacity (TAC) of water extracts (WE), alkaline extracts before (AlE) and after acidification (AcE) and combined hydrolysates (AAH), determined by the FC method, expressed in µg of gallic acid per g of investigated soil dry mass.

Land use	Soil sample	WE	AlE	AcE	HA share of AlE [%]	AAH
	TAC ± SD [µg g^−1^ d.m.]					
Fallows (O)	O1	21.25 ± 3.44	994.00 ± 39.60	408.00 ± 16.97	58.95 ± 1.64	15,222.67 ± 681.57
	O2	11.09 ± 2.90	1177.00 ± 142.52	171.50 ± 7.78	85.43 ± 1.49	8088.00 ± 498.83
	O3	7.63 ± 0.87	178.33 ± 19.09	83.33 ± 17.00	53.27 ± 2.99	10,172.00 ± 748.29
	O4	5.08 ± 1.96	291.67 ± 13.44	84.00 ± 5.66	71.20 ± 0.53	7504.00 ± 630.52
	**O (mean)**	**11.26 ± 7.09**^**a**^	**660.25 ± 498.84**^**a**^	**186.70 ± 153.23**^**a**^	**65.99 ± 14.11**^**a**^	**10,246.67 ± 3509.43**^**a**^
Farmfields (P)	P1	15.95 ± 0.97	850.67 ± 38.89	289.33 ± 35.36	88.97 ± 6.33	6368.00 ± 195.16
	P2	11.95 ± 0.59	766.00 ± 14.14	84.50 ± 3.54	52.92 ± 0.26	2052.00 ± 153.73
	P3	7.35 ± 0.71	239.33 ± 44.11	112.67 ± 17.21	59.75 ± 6.70	8873.33 ± 370.61
	P4	6.61 ± 2.71	135.00 ± 15.56	54.33 ± 8.62	57.46 ± 3.08	7376.00 ± 595.76
	**P (mean)**	**10.47 ± 4.35**^**a**^	**497.75 ± 362.80**^**a**^	**135.20 ± 105.48**^**a**^	**65.22 ± 15.02**^**a**^	**6167.33 ± 2930.27**^**a**^
Forests (L)	L1	114.05 ± 1.81	1263.00 ± 85.56	537.33 ± 55.15	62.83 ± 0.75	13,598.00 ± 828.73
	L2	64.85 ± 4.91	1530.43 ± 127.14	532.33 ± 8.49	46.23 ± 7.18	15,984.00 ± 248.90
	L3	24.90 ± 3.39	1944.33 ± 128.47	722.67 ± 12.73	58.95 ± 5.68	17,890.67 ± 1679.32
	L4	28.79 ± 4.54	2228.67 ± 183.85	1198.33 ± 120.9	57.43 ± 6.65	14,549.33 ± 922.07
	**L (mean)**	**58.15 ± 41.38**^**a**^	**1741.60 ± 428.95**^b^	**747.65 ± 313.23**^**b**^	**53.27 ± 7.10**^**a**^	**15,505.50 ± 1868.22**^**b**^

Results are expressed as mean values ± standard deviation (*n* = 3). Letters a and b indicate significant differences between mean TAC values for each extract type, within studied land type groups (p < 0.05). Significant values are in bold.

Humic substances (humic acids—HA, fulvic acids—FA and humins—HU), present in the soil are the most reactive part of organic matter^41^. They are complex and heterogeneous mixtures of polydispersed materials formed by biochemical and chemical reactions during the decay and transformation of plant and microbial remains^18^. These organic molecules contain phenolic electron-donating moieties, due to which they may act as antioxidants^42^, thus significantly contributing to TAC and influencing soil quality. To determine the share of HA in the total antioxidant potential of the studied soils, the previously obtained alkaline extracts were acidified to precipitate humic acids. HA were discarded and the resultant mixtures (AcE) containing the fraction of dissolved phenols together with fulvic acids were again subjected to the FC test. Owing to that, it was possible to estimate the contribution of the HA fraction to the total antioxidant potential of the sample after alkaline extraction. It was calculated as a difference in the antioxidant capacity of the alkaline extracts and the same extracts after acidification, and expressed as a percentage share of the total antioxidant capacity of the AlE, ranging from 46 to 89% (Table 3). The highest mean HA share of TAC was determined for abandoned fallows and farmlands (65–66%), while in the case of forest soils it was equal to 53%, but the differences within the studied groups were not statistically significant. Nevertheless, the slightly lower mean value of the parameter obtained for the forest soils can be explained by a constant inflow of fresh organic matter in the organic horizons, which may increase the share of material with a low humification degree^43^. The antioxidant properties of HS were also investigated by Aeschbacher et al^42^., who evaluated the electron-donating capacities of humic substances and correlated them with their phenolic moieties formed from higher plant precursors such as lignin and tannins. The presented results indicate that humic acids may contribute to over 50% of the TAC of soils, irrespective of the land use type.

**Figure 1:**
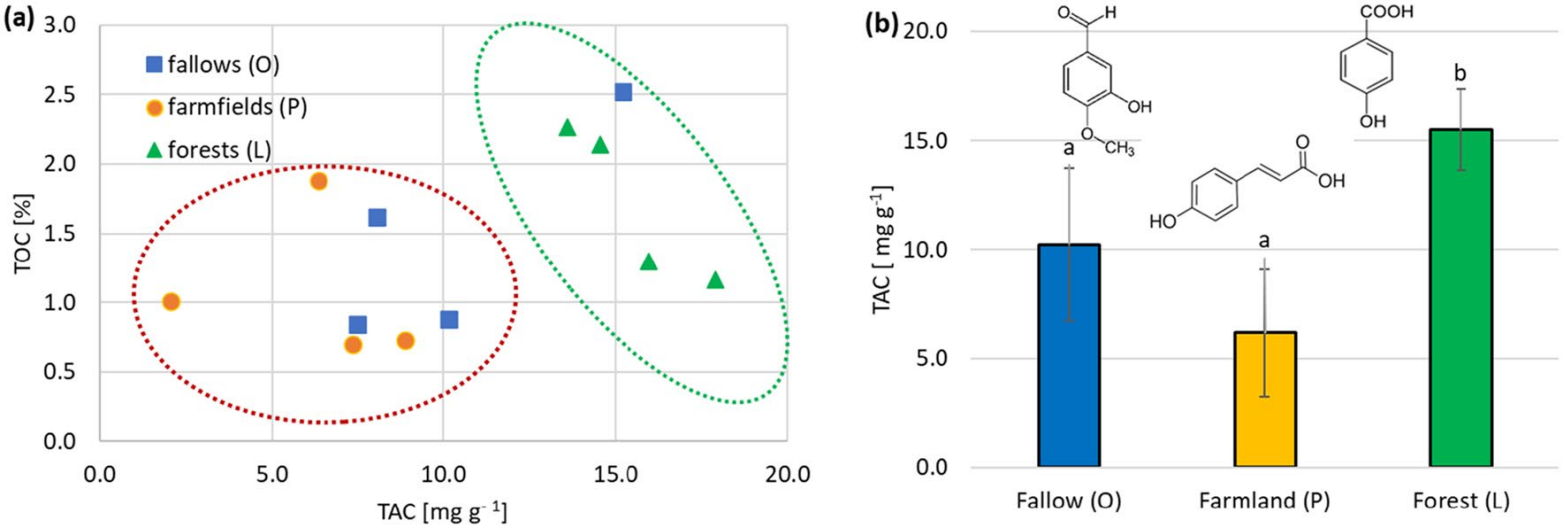
Dependence of organic carbon content (TOC) in the A horizon of the studied soils on their antioxidant potential (TAC), estimated in AAH extracts (**a**). Summary of the mean TAC concentrations in the soil materials from surface horizons of abandoned fallows (O), farmlands (P), and forest (L) soils (**b**). Results are expressed as mean values ± standard deviation (*n* = 3). Letters a and b indicate significant differences between mean TAC values for each extract type, within studied land type groups (p < 0.05).

The last type of soil extract analyzed for the total antioxidant potential (FC assay) was obtained by the acid hydrolysis and alkaline re-hydrolysis of the soil samples (AAH) (Table 3). The average antioxidant capacity of the AAH samples was the highest for the A horizons of forest soils (15,505.50 µg g^−1^). Abandoned fallows and cultivated soils exhibited significantly lower mean TAC levels, with 10,246.67 and 6334.0 µg g^−1^ of soil dry mass, respectively (Fig. 1b). This is in line with the trends observed for alkaline extracts (AlE) of the tested soils. The extraction method based on acid–base hydrolysis was the most efficient one among the tested variants. It released the largest pool of compounds, capable of reacting with the FC reagent, from the studied soil materials. The measured TAC values were on average 5 to even 50 times higher than those obtained in AlE and AcE extracts. This is due to the use of very high (10 M) concentrations of sodium hydroxide and hydrochloric acid, which enforced the hydrolysis of the ester and glycosidic bonds, strongly retaining phenolic acids in the soil matrix^24^. The extraction efficiency was also enhanced by microwave radiation, which supported the acid hydrolysis and significantly shortened this step compared to the original procedure^24^.

The presented results demonstrate that various pools of antioxidants can be isolated from soils, depending on the extraction efficiency of the solvent used. The highest TAC values, measured with the FC assay, were determined in the forest A horizons, irrespective of the soil extraction type. This was attributed to the highest input of fresh organic matter in forests, which is the source of secondary plant metabolites. The measured antioxidant capacities were compiled with the total organic carbon contents of the investigated soils (Fig. 1a). Significant (p < 0.05), moderately positive correlation coefficients ranging from 0.452 for AAH extracts up to 0.586 for AcE mixtures were found (Supplementary Table S1), being in line with the findings of Rimmer and Smith^14^. The soil materials taken from abandoned fallows and farmfields were characterized by significantly lower TAC than the samples obtained from the A horizons of forest soils (Fig. 1b).

When comparing the extraction methods used in this study, it can be concluded that alkaline extraction (0.1 M NaOH) is not an efficient method for the quantification of antioxidant compounds in soil. Nevertheless, it reflects the general trend in the content of phenolic compounds in the analyzed soils. It can therefore serve as a preliminary method for the determination of the antioxidant potential of soil samples before the more efficient acid–base hydrolysis and a subsequent chromatographic identification of the extracted compounds.

### Phenolic profiles in soils under various land use.

The quantitative and qualitative results of the GC–MS analysis of the studied AAH extracts within each of the analyzed land use groups are presented in Table 4 and Supplementary Figure S2. In the combined mixtures from acid hydrolysis and alkaline re-hydrolysis of soil samples, the following nine compounds were identified: p-Hydroxybenzoic acid (p-HA), Suberic acid (SA), Isovanillic acid (IVA), Homovanillic acid (HVA), Azelaic acid (AZA), Protocatechuic acid (PA), Syringic acid (SYR), Esculetin (ESC) and p-Coumaric acid (p-CO). Most of them have literaturally reported antioxidant capacities, although not all the compounds belong to the phenolics group. One of such chemicals is suberic (octanedioic) acid, commonly present in the bark of some tree species, such as birch^44^, exhibiting a significant share in the studied soil extracts. It is a fatty-acid derivative originating from plant biomacromolecules (cutin and suberin), classified as a recalcitrant aliphatic organic matter fraction^45^. Due to its insoluble and non-hydrolysable nature, it tends to accumulate in soil, enriching SOM fractions. Another representative of this group found in the studied topsoils was azelaic acid. This 9-carbon atom dicarboxylic acid is produced by a variety of plants, commonly wheat species, in response to biotic and abiotic stress conditions^46^, therefore having antioxidant properties.

**Table 4:** Qualitative and quantitative (in µg g^−1^ of dry soil mass) characteristic of individual compounds in the AAH extracts of abandoned soils, farmlands, and forest soils.

	Abandoned fallows				Farmlands				Forests			
	Mean	Min	Max	SE*	Mean	Min	Max	SE	Mean	Min	Max	SE
	(µg g^−1^)				(µg g^−1^)				(µg g^−1^)			
p-HA	22.96	21.11	24.05	0.65	140.31	138.57	142.73	1.25	607.47	389.45	745.22	78.83
SA	11.42	6.89	21.46	3.37	10.33	10.15	10.57	0.12	505.14	400.69	691.09	65.18
IVA	11.90	6.30	14.51	1.94	39.71	39.07	40.54	0.43	1687.02	1521.18	2301.84	284.16
HVA	2.51	1.96	2.93	0.23	1.69	1.67	1.72	0.01	20.55	7.11	44.24	8.23
AZA	14.34	7.18	31.94	5.89	19.30	16.64	19.06	0.17	1074.07	683.00	1676.31	242.99
PA	0.78	0.55	1.24	0.16	2.67	2.60	2.75	0.04	87.12	50.52	147.21	22.93
SYR	2.87	2.19	4.33	0.49	9.64	9.55	9.76	0.07	31.23	16.56	38.66	5.09
ESC	20.04	3.64	68.94	16.30	20.93	20.59	21.37	0.23	244.01	92.57	436.84	71.70
p-CO	5.95	5.00	6.88	0.47	4.11	4.07	4.18	0.03	158.26	78.95	266.57	42.19
SUM	92.77	61.21	165.54	24.39	248.70	245.33	253.26	2.37	4341.07	4137.45	4572.39	91.65

*SE*—*standard error.

**Figure 2:**
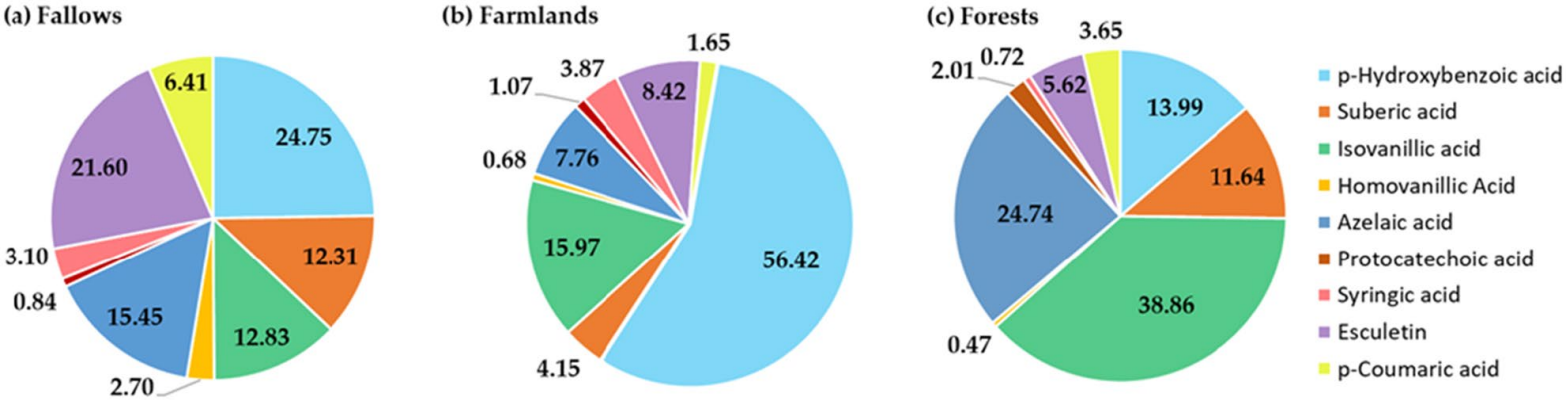
The mean percentage share of individual compounds identified in AAH extracts from investigated abandoned fallows (**a**), farmlands (**b**), and forest soils (**c**).

Among the phenolic compounds identified in soil extracts, there were representatives of vanilyl (IVA, HAV), syringyl (SYR), as well as hydroxycinnamic (p-CO, ESC) acid derivatives (Table 4, Figure S2). These three groups of phenolic compounds content and ratios in lignins are plant species-specific^19,43,47^, which was reflected in their mutual percentage share in the soils under various land uses (Fig. 2). Although the studies on the phenolic profiles of soil are still limited, the results obtained here are in accordance with the available literature findings^14,18,19^, where the same or similar compounds belonging to the three PCs groups were reported.

There was considerable variation in the content of the respective phenolic and non-phenolic compounds identified in the AAH soil extracts. In general, the highest contents of antioxidants were found in the forest soils (Table 4), achieving an average of 4341.07 µg g^−1^ of soil dry mass, which was over 17 and 47 times higher than its mean value for farmlands (248.70 µg g^−1^ d.m.) and fallows (92.77 µg g^−1^ d.m.), respectively. When compared with the literature, the sum of compounds determined in forest soils was in the same range as the content of phenolics in the plant material of meadows (3204.72–5736.45 µg g^−1^ d.m.)^19^. What is more, the sum of compounds found in the topsoils of investigated fallows under meadow vegetation was significantly lower than the amounts estimated by Ziółkowska et al^24^. This might be due to the differences in the chemical composition of the extracts, which is highly plant-specific and may also arise from the specificity of the chromatographic method utilized in the studies (GC–MS vs LC–MS).

It is also worth emphasizing that the sum of compounds in AAH extracts estimated with the GC–MS technique (SUM) was much lower than the total antioxidant capacity (TAC) assessed in the FC test (Table 3), although the main trends observed within the land use groups were maintained. This corroborates the observations made by other authors^28,33,34,48^, who claim that the FC method is useful in assessing the total antioxidant potential of the sample (including the share of non-phenolic redox-active compounds) rather than solely the total phenolic compounds content. Therefore, FC assay should be coupled with the phenolic profiling to verify the actual contribution of PCs in the antioxidant properties of soils^49^.

Generally, in investigated abandoned fallow soils the highest share in the sum of identified compounds constituted p-hydroxybenzoic acid (24.75%) and coumarin derivative esculetin with p-coumaric acid, comprising a total of 28.01% (Fig. 2). The highest p-HA share coincides with the studies of Rimmer and Abbott^20^, who found that it was one of the dominant phenolics in the subset of surface soil samples under various land uses, including semi-natural soils and pastures. Esculetin (21.60% of the SUM) is a polyphenol present in many medicinal herbal plants, such as various Artemisia species^50,51^, which were also found in the vegetation profile of the studied fallows (Table 1). It has been reported to exert strong anti-proliferative and antioxidant activities^50^. In the undisturbed ecosystem of abandoned fallows, there was also an accumulation of aliphatic organic matter fraction (sum of SA and AZA) observed, which contributed up to 27.81% of the identified compounds. Isovanillic acid amounted to 12.83%, whilst syringic and protocatechoic acids constituted less than 5% of the SUM.

In the cultivated soils, similarly to fallows, p-hydroxybenzoic acid was dominant, constituting 56.42% of the compounds sum. This is in agreement with the findings of several studies performed on arable lands, where p-HA was identified as the most abundant phenolic compound in the field soil samples^2,52,53^. Also, Zhou et al^54^. noticed that p-HA was one of the main PCs in rhizospheric soil under a continuous mono-cropping system. The greatest concentration of p-HA in the arable soils of this study can be attributed to its production by microbial synthesis^55^. This is due to the regular input of post-harvest residues on soil surface, which enhances microbial activity and results in the overall highest share of p-hydroxybenzoic acid^31^ in SUM. Isovanillic acid constituted 15.97% of SUM. It is a product of the microbial degradation of lignin, presumably present in woody post-harvest residues in investigated arable soils. Aliphatic acids (SA and AZA) were in the minority (11.91% of SUM), contrary to their content in fallow or forest soils. Similarly, esculetin abundance was decreased in farmland soils (8.42%), which is in line with the very limited and random occurrence of herbal-type vegetation on cultivated plots.

According to the results obtained, isovanillic acid was the predominant phenolic in the extracts of forest A horizons, constituting 38.86% of the extracted compounds pool (Fig. 2). This is in line with the studies of Rimmer and Abbott^20^, who observed the highest concentration of IVA in woodland soils. Also, Dębska and Banach-Szott^56^ demonstrated considerably higher amounts of vanilyl compounds in the mineral horizons of forest soils, with respect to syringyl and cinnamyl derivatives. p-HA share was much lower here (13.99%), compared to fallow and cultivated soils. Due to the literature, IVA and p-HA are common products of lignin biodegradation^57^ in conifer forests. They are products of veratric and p-anisic acid demethylation and hydroxylation of the latter by the specific bacteria strains growing on the wood flour^58^. Their concentration considerably increases in litters with biodegradation progress^59^. In the extracts of forest soils there were also significant amounts of azelaic and suberic acids, reaching on average 24.74 and 11.64% of the chemicals sum, respectively. They originate from the cuticle that seals the aerial epidermis, and suberin that is present in the periderm of barks and underground organs of trees^60^. Hydroxycinnamic acid derivative p-coumaric acid and esculetin were in the minority (3.65% and 5.62%, respectively).

**Figure 3:**
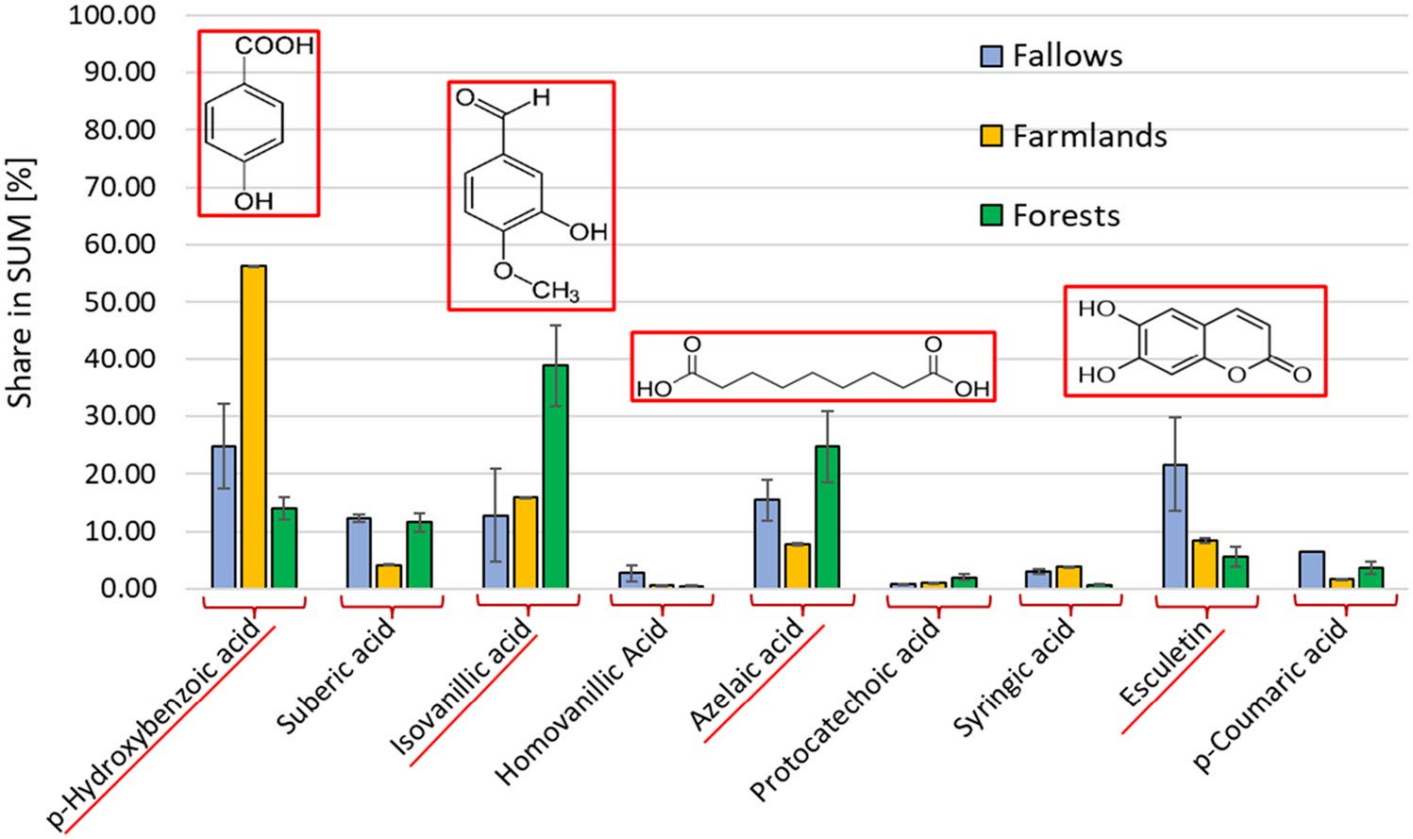
The comparison of mean percentage shares of individual compounds identified in AAH extracts from investigated abandoned fallows, farmlands, and forest soils. Results are expressed as mean values ± standard deviation (*n* = 3).

Considering the mutual share of the compounds identified in the AAH extracts (Fig. 3), it can be concluded that the vegetation, characteristic of the land use type, may be the predominant factor influencing the phenolics profile in the studied soils. However, more extensive research is necessary to fully verify the synergistic effect of land use and soil typology on the antioxidants content of soils. p-Hydroxybenxoic acid, isovanillic acid and esculetin were the most abundant phenolics in the studied A horizons of soils in the temperate climate zone. However, one should not underestimate the significant share of aliphatic organic matter constituents (suberic and azelaic acids) in the studied topsoils. Therefore, further studies to clarify their role in the antioxidant capacity of soils should be evaluated.

## Conclusions

The presented results verify and significantly complement the existing literature findings on the phenolic and non-phenolic compounds content in soils in temperate climate zone. The highest total antioxidant capacity was assigned to woodland A horizons. Cultivated soils and abandoned fallows were characterized by similar and much lower TAC values than the forest soils, regardless of the extract type. Acid hydrolysis combined with alkaline re-hydrolysis released the biggest pool of redox-active compounds, from soil materials among the tested extraction variants. The antioxidant capacity of the studied soils was positively correlated with their organic carbon content. It was also demonstrated that humic acids contribute to over 50% of TAC in alkaline soil extracts, irrespective of land use.

The estimated phenolic profiles in soils were vegetation specific. The major compounds identified in soils were p-hydroxybenzoic acid, isovanillic acid and esculetin. p-HA dominated in soils taken out of cultivation, whereas IVA prevailed in forest A horizons. Herbaceous plants in abandoned fallows were presumably the source of esculetin, which together with p-HA were the main PCs in set-aside soils. There were also considerable amounts of aliphatic fatty acids (azelaic and suberic acids) found in the studied topsoils, whose role in redox processes should be further evaluated.

### Supplementary Information

Supplementary Information.

## Data Availability

Data will be made available on reasonable request.
