# Supplementary material for: Antioxidant capacity sources of soils under different land uses
Source: Sci Rep. 2024 Apr 10;14:8394. doi: 10.1038/s41598-024-58994-9 (PMC11006951; doi:10.1038/s41598-024-58994-9)
Supplement: Supplementary file 1 — Supplementary Information. [file 41598_2024_58994_MOESM1_ESM.docx]

Antioxidant capacity sources of soils under different land uses

Irmina Ćwieląg-Piasecka ^1^*, Jacek Łyczko ^2^, Elżbieta Jamroz ^1^, Andrzej Kocowicz ^1^ and Dorota Kawałko^1^

^1^ Wroclaw University of Environmental and Life Sciences, Institute of Soil Science, Plant Nutrition and Environmental Protection, Grunwaldzka 53 St., 50-357 Wroclaw, Poland; [irmina.cwielag-piasecka@upwr.edu.pl](mailto:irmina.cwielag-piasecka@upwr.edu.pl), [jacek.lyczko@upwr.edu.pl](mailto:jacek.lyczko@upwr.edu.pl), [elzbieta.jamroz@upwr.edu.pl](mailto:elzbieta.jamroz@upwr.edu.pl), [andrzej.kocowicz@upwr.edu.pl](mailto:andrzej.kocowicz@upwr.edu.pl), [dorota.kawalko@upwr.edu.pl](mailto:dorota.kawalko@upwr.edu.pl)

^2^ Wrocław University of Environmental and Life Sciences, Department of Food Chemistry and Biocatalysis, Norwida 25, 53-375 Wrocław, Poland

*Corresponding author

**Supplementary information**


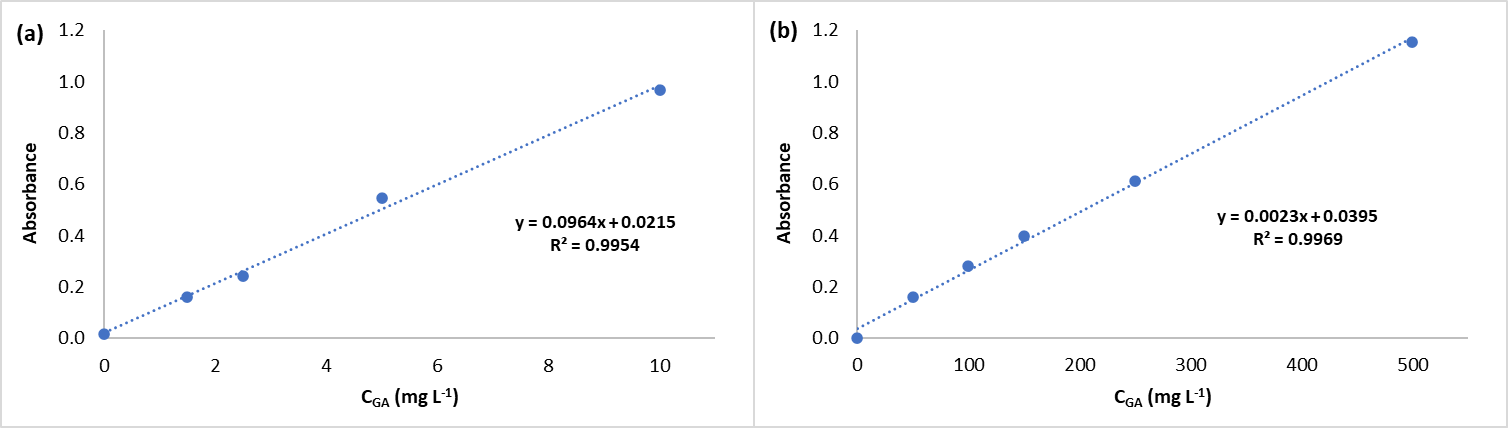


**Figure S1** Calibration curve for the assessment of total antioxidant activity using Folin- Ciocâlteu method, expressed in gallic acid equivalents (C_GA_ in mg L^–1^), for (a) water extracts (WE), (b) other extract types analyzed in the study (AlE, AcE, AAH).

**Table S1** Correlation coefficients between TOC of soils and TAC values of various extracts (WE, AlE, AcE, AAH) types. Marked * correlation coefficients are significant with p < 0.05 (N=12).

| Variable | **We** | **AlE** | **AcE** | **AAH** | **TOC** | **SUM** |
| --- | --- | --- | --- | --- | --- | --- |
| **We** | - | 0.433* | 0.429* | 0.465* | 0.531* | 0.669* |
| **AlE** |  | - | 0.909* | 0.646* | 0.550* | 0.837 |
| **AcE** |  |  | - | 0.726* | 0.586* | 0.840 |
| **AAH** |  |  |  | - | 0.452* | 0.755* |
| **TOC** |  |  |  |  | - | 0.407* |

TOC—total organic carbon, TAC—total antioxidant activity, WE—water extracts, AlE—alkaline extracts, AcA—alkaline extracts after humic acids removal, AAH—mixed extracts obtained in acid hydrolysis and alkaline rehydrolysis, SUM – sum of the phenolic and non-phenolic compounds identified with GC-MS.


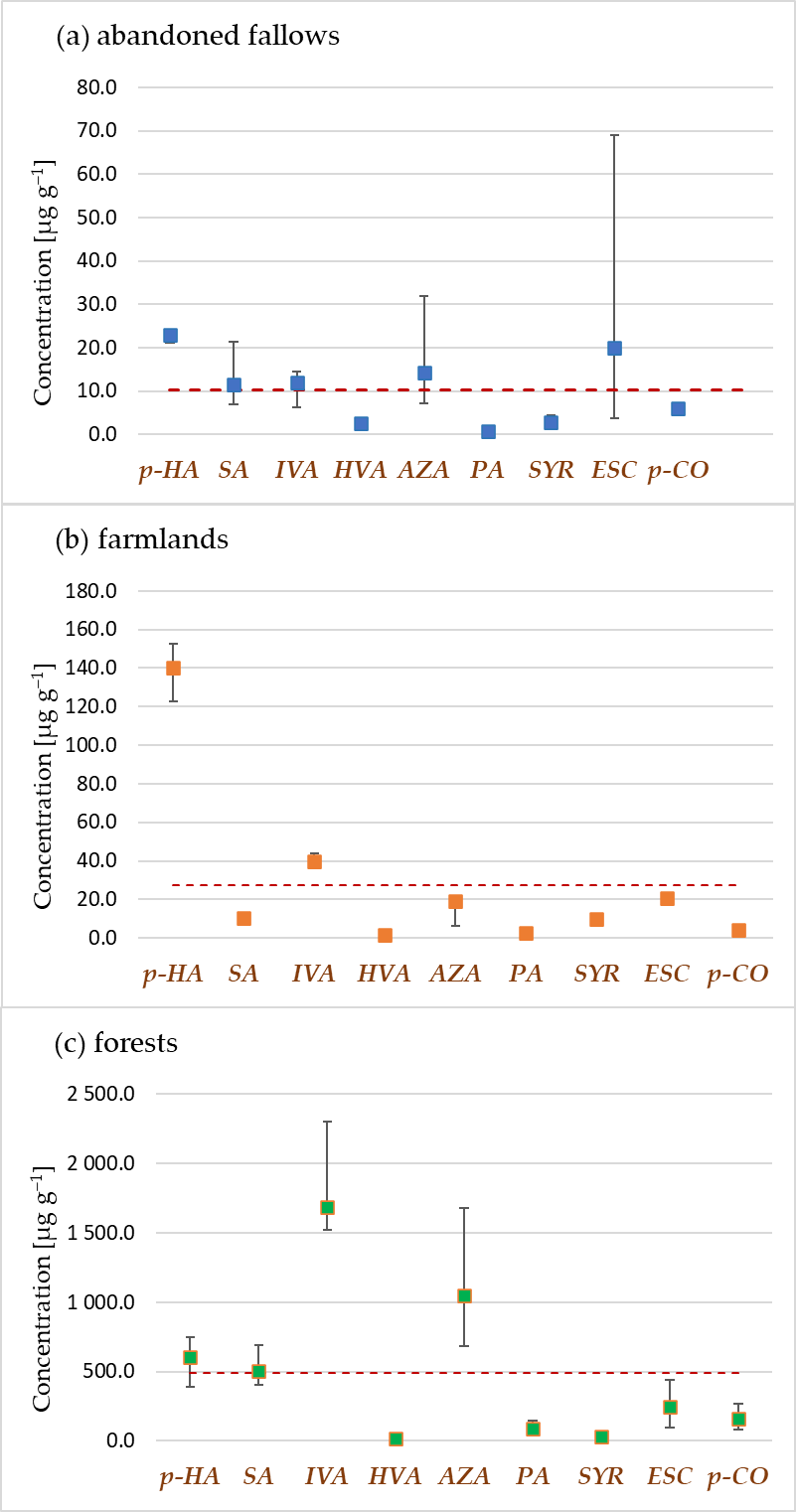


**Figure S2.** Mean concentration values of the phenolic and non-phenolic compounds extracted from (a) abandoned fallows, (b) farmlands, (c) forests soils. The red dotted line within each figure marks the mean concentration of the identified compounds sum. Whiskers indicate the minimum and maximum concentrations.
